# Characteristics of Misclassified CT Perfusion Ischemic Core in Patients with Acute Ischemic Stroke

**DOI:** 10.1371/journal.pone.0141571

**Published:** 2015-11-04

**Authors:** Ralph R. E. G. Geuskens, Jordi Borst, Marit Lucas, A. M. Merel Boers, Olvert A. Berkhemer, Yvo B. W. E. M. Roos, Marianne A. A. van Walderveen, Sjoerd F. M. Jenniskens, Wim H. van Zwam, Diederik W. J. Dippel, Charles B. L. M. Majoie, Henk A. Marquering, MR CLEAN trial investigators()

**Affiliations:** 1 Dept. of Biomedical Engineering and Physics, Academic Medical Center, Amsterdam, The Netherlands; 2 Dept. of Radiology, Academic Medical Center, Amsterdam, The Netherlands; 3 Dept. of Neurology, Academic Medical Center, Amsterdam, The Netherlands; 4 Dept. of Radiology, Leiden University Medical Center, Leiden, The Netherlands; 5 Dept. of Radiology, Radboud University Medical Center, Nijmegen, The Netherlands; 6 Dept. of Radiology, Maastricht University Medical Center+, Maastricht, The Netherlands; 7 Dept. of Neurology, Erasmus Medical Center, Rotterdam, The Netherlands; Fraunhofer Research Institution of Marine Biotechnology, GERMANY

## Abstract

**Background:**

CT perfusion (CTP) is used to estimate the extent of ischemic core and penumbra in patients with acute ischemic stroke. CTP reliability, however, is limited. This study aims to identify regions misclassified as ischemic core on CTP, using infarct on follow-up noncontrast CT. We aim to assess differences in volumetric and perfusion characteristics in these regions compared to areas that ended up as infarct on follow-up.

**Materials and Methods:**

This study included 35 patients with >100 mm brain coverage CTP. CTP processing was performed using Philips software (IntelliSpace 7.0). Final infarct was automatically segmented on follow-up noncontrast CT and used as reference. CTP and follow-up noncontrast CT image data were registered. This allowed classification of ischemic lesion agreement (core on CTP: rMTT≥145%, aCBV<2.0 ml/100g and infarct on follow-up noncontrast CT) and misclassified ischemic core (core on CTP, not identified on follow-up noncontrast CT) regions. False discovery ratio (FDR), defined as misclassified ischemic core volume divided by total CTP ischemic core volume, was calculated. Absolute and relative CTP parameters (CBV, CBF, and MTT) were calculated for both misclassified CTP ischemic core and ischemic lesion agreement regions and compared using paired rank-sum tests.

**Results:**

Median total CTP ischemic core volume was 49.7ml (IQR:29.9ml-132ml); median misclassified ischemic core volume was 30.4ml (IQR:20.9ml-77.0ml). Median FDR between patients was 62% (IQR:49%-80%). Median relative mean transit time was 243% (IQR:198%-289%) and 342% (IQR:249%-432%) for misclassified and ischemic lesion agreement regions, respectively. Median absolute cerebral blood volume was 1.59 (IQR:1.43–1.79) ml/100g (P<0.01) and 1.38 (IQR:1.15–1.49) ml/100g (P<0.01) for misclassified ischemic core and ischemic lesion agreement, respectively. All CTP parameter values differed significantly.

**Conclusion:**

For all patients a considerable region of the CTP ischemic core is misclassified. CTP parameters significantly differed between ischemic lesion agreement and misclassified CTP ischemic core, suggesting that CTP analysis may benefit from revisions.

## Introduction

Acute ischemic stroke is the third most common cause of death and the leading cause of permanent invalidity in industrialized countries [1]. Early recanalization of arteries to restore perfusion of regions at risk but still viable (penumbra) is vital [2]. Recanalization can be achieved by intravenous administration of recombinant tissue plasminogen activator (rtPA) or intra-arterial treatment (IAT) [3]. Intravenous rtPA is proven to be a beneficial treatment for ischemic stroke patients, but due to haemorrhage risk, has a limited time-window of four and a half hours after onset of symptoms [4]. Recent multi-center randomized control trials, like MR CLEAN, EXTEND-IA, SWIFT PRIME, ESCAPE and REVASCAT, showed that intra-arterial treatment after intravenous rtPA is beneficial in restoring reperfusion within 6 hours after onset of symptoms and significantly improved patient outcome after 90 days [5–9]. However, a successful early recanalization by IAT does not guarantee a good outcome at 90 days. CT perfusion (CTP) has the potential to limit the number of futile recanalizations by improving critical patient selection for treatment [10,11].

CTP software distinguishes ischemic core from penumbra and unaffected regions by measuring blood perfusion in cerebral regions, based on venous contrast agent injection. Cerebral Blood Flow (CBF), Cerebral Blood Volume (CBV), and Mean Transit Time (MTT) are parameters determined by CTP analysis, which are used for assessment of ischemic core and penumbra [12–14]. CTP has certain advantages [15–18]. It is widely available at almost all emergency units, rapid and easily performed, and has a high spatial resolution [12]. Therefore this technique has the potential to become a useful diagnostic tool in acute settings.

Despite the potential of CTP there are some issues which may cause inaccurate results and hamper its acceptance in clinical practice [16]. Different processing software packages, based on different underlying algorithms, have been proven to produce different results [19,20]. Other causes can be incorrect manual selection of arterial or venous input or too short acquisition times causing truncation of time attenuation curves [21–23]. Head-movement or biological (certain vascular- or neurodiseases or cardiac output) or anatomical (lesions, variations in Circle of Willis or vascular system) causes can also influence data analysis [22–24].

CTP software creates summary maps to visualize ischemic core and tissue “at-risk” (penumbra). Classification of this ischemic core and penumbra is based on parametric thresholds. Suboptimal thresholds may influence CTP results. Thresholds used for classification of ischemic core are derived from large samples and can agree in large-scale studies. However, these thresholds can produce large errors when applied to individual patients [22,25]. Studies have shown that strict threshold values can lead to over- or underestimation of ischemic core volume and penumbra, as they may not be specific for individual cases [23,26]. A previous study showed the appearance of false ischemic penumbra, caused by upstream flow restriction, old infarction, vascular anatomy variations and dysregulation, and patient misplacement in scanner [27].

Currently CTP still misclassifies a large area as ischemic core [28]. For CTP to be more reliable in clinical settings, accuracy has to be improved [1,17]. MR-DWI is considered the most accurate modality to assess cerebral infarct and has been used as a gold standard in various studies [29,30]. However, MR-DWI is not commonly available in the acute setting of treatment of ischemic stroke patients [1]. Alternatively, it is proposed to use follow-up noncontrast CT as reference standard. Follow-up noncontrast CT is accurate in quantification of final infarct [31]. However, it is known that in the period between baseline and follow-up scanning, the infarct may grow [32,33]. Nevertheless, because it is not possible to have an ischemic core on baseline that has disappeared on follow-up imaging, follow-up noncontrast CT can be used as reference standard to identify regions misclassified as ischemic core on CTP. In this study we aim to identify misclassified CTP ischemic core using follow-up noncontrast CT and determine perfusion characteristics of ischemic core that can be used to improve CTP analysis.

## Materials and Methods

### Population

This study included patients from the MR CLEAN trial (www.mrclean-trial.org) [5]. Patients were eligible for inclusion in the MR CLEAN trial, if they had a clinical diagnosis of acute ischemic stroke, with a deficit on the National Institutes of Health Stroke Scale (NIHSS) of 2 points or more, CT or MRI ruling out haemorrhage, and intracranial artery occlusion of the distal intracranial carotid artery or middle (M1/M2) or anterior (A1/A2) cerebral artery. All patients or relatives signed an informed consent.

Seventy-one patients from MR CLEAN trial received >100 mm brain coverage baseline CTP. Final infarct volume was determined on 5–7 day follow-up noncontrast CT. For two patients, 5–7 day CT was not available and 24-hours follow-up noncontrast CT was used for determining final infarct volume. Patients were excluded for the following reasons: CTP available, but no follow-up CT (N = 18); excessive movement (N = 2); poor quality of the scans (N = 4); midline shift over 5 mm on follow-up CT (N = 7); craniectomy on follow-up CT (N = 3); no ischemic core on baseline CTP (penumbra only) (N = 2). After exclusion, final sample size of this study was 35 patients.

### CT imaging

#### Baseline CTP imaging

Scanner, scanning protocol and acquisition time differed per center; detailed information can be found in Table 1. If necessary, multiple 0.5 mm slices were averaged to 5 mm slices using MATLAB (The MathWorks, Massachussets, USA) to enable processing by the perfusion analysis software. CTP series were post-processed using commercially available Philips IntelliSpace Portal 7.0, Brain Perfusion application software (Royal Philips Healthcare, Best, The Netherlands). 3D motion correction and filtering was applied on all scans, using the built-in features. To ensure standard protocol, arterial input function (AIF) and venous output function (VOF) were always placed in the internal carotid artery and sagittal sinus. Ischemic core, defined as a relative MTT ≥ 145% and an absolute CBV < 2.0 ml/100g, was automatically classified and presented in a summary map [25]. All quantitative (absolute) parameter maps (aCBF, aCBV, aMTT) and summary maps were exported for further analysis. Relative parameter values were calculated in respect to their contralateral value resulting in relative MTT (rMTT), CBF (rCBF), and CBV (rCBV) in MATLAB.

**Table 1:** Scanners, brain coverage, acquisition time and contrast agent for CT perfusion amongst hospitals included in this study.

Center	1	2	3	4
**Scanner**	Toshiba Aquilion ONE^a^	Toshiba Aquilion ONE^a^	Siemens Somatom Definition Flash^b^	Siemens Somatom Definition Edge^b^
**Kernel**	FC26	FC26	H31s	H31s
**Nr. of patients**	13	6	14	2
**Brain coverage**	160 mm	160 mm (except 1: 140 mm)	100 mm	125 mm or 155 mm
**Acquisition time**	53 sec. (N = 13)	53 sec. (N = 6) or 50 sec. (N = 1)	44 sec. (N = 5) or 60 sec. (N = 9)	53 sec. (N = 1) or 60 sec. (N = 1)
**Contrast agent**	Ultravist 370	Iomeprol 300	Ultravist 300	Iomeprol 400
**Contrast volume**	50 ml followed by 50 ml saline	50 ml followed by 40 ml saline	50 ml followed by 40 ml saline	40 ml followed by 45 ml saline
**Injection rate**	5 ml/sec.	5 ml/sec.	7 ml/sec.	6 ml/sec.
**Start of acquisition**	5 sec. after start of injection	5 sec. after start of injection	2 sec. after start of injection	With start of injection

^a^ Toshiba Medical Systems, Tokyo, Japan

^b^ Siemens, Erlangen, Germany

It has been shown that truncation may cause inaccuracies in CTP results [21]. AIF, VOF and tissue time-attenuation curves (TAC) were visually classified as truncated or complete. Attenuation curves were classified as complete when the attenuation values returned close to their baseline values

#### Follow-up noncontrast CT imaging

Patients received follow-up noncontrast CT at 24 hours and 5–7 days after hospitalization. Final infarct size was determined on 5–7 day follow-up noncontrast CT scanning if possible. When these were not available, infarct was determined on 24 hours follow-up noncontrast CT (N = 2). Follow-up noncontrast CT images were registered with motion corrected CTP images using Elastix [34]. Infarct on follow-up noncontrast CT was determined using a semi-automated method by in-house developed software (MATLAB) [35].

#### Ethics statement

The CTP protocol has been approved by the institutional review board (Medisch Ethische Toetsings Commissie) from the Academic Medical Center, Amsterdam, The Netherlands. Patients or legal representatives signed informed consent.

### Selection of misclassified ischemic core

**Fig 1:**
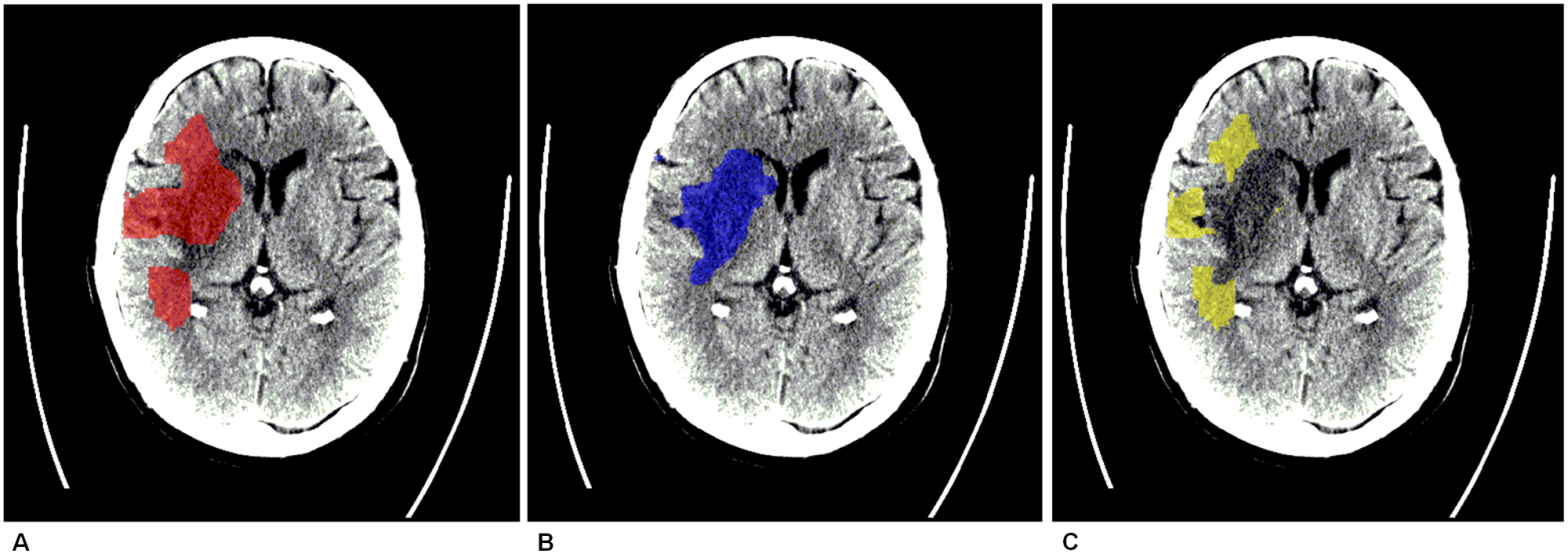
Example of a misclassified ischemic core. A. Ischemic core as selected by CT perfusion software on baseline CTP (projected on follow-up noncontrast CT). B. Final infarct as determined on follow-up noncontrast CT. C. Misclassified ischemic core region projected on follow-up noncontrast CT.

Registration of follow-up noncontrast CT with baseline CTP allowed direct comparison of CTP derived ischemic core and follow-up noncontrast CT derived infarct (Fig 1) CTP ischemic core, follow-up noncontrast CT infarct and CTP derived parameter maps registration was performed in MATLAB.

Voxel-based comparison of CTP ischemic core with follow-up noncontrast CT infarct resulted in two areas of interest. The first is “misclassified ischemic core”, which is defined as the area classified as ischemic core on CTP and not as infarct on follow-up noncontrast CT. The second area of interest is the area in which CTP ischemic core and follow-up noncontrast CT infarct agree, which is referred to as “ischemic lesion agreement” in the following.

Misclassified ischemic core and ischemic lesion agreement were used as regions of interest (ROIs) for further analysis. ROIs were projected on follow-up noncontrast CT for visual assessment (Fig 1) and on quantitative parameter maps of the MTT, CBF, and CBV.

### Statistical Analysis

Median and IQR of the total ischemic core, misclassified ischemic core and ischemic agreement lesion volume were determined. The false discovery ratio (FDR), defined as the misclassified ischemic core volume divided by the total ischemic core volume, was calculated. Median values of voxel-based CTP parameter values (rMTT, aCBF, rCBF, aCBV, and rCBV) were calculated per patient. Median of these median parameter values for misclassified ischemic core and ischemic lesion agreement amongst all patients were combined and analyzed separately with a rank-sum test. All analyses and visualization were performed in IBM SPSS 22.0 (IBM Corporation, Armonk, New York, USA).

## Results

### Core volumes, false discovery ratio & identification of truncation

**Fig 2:**
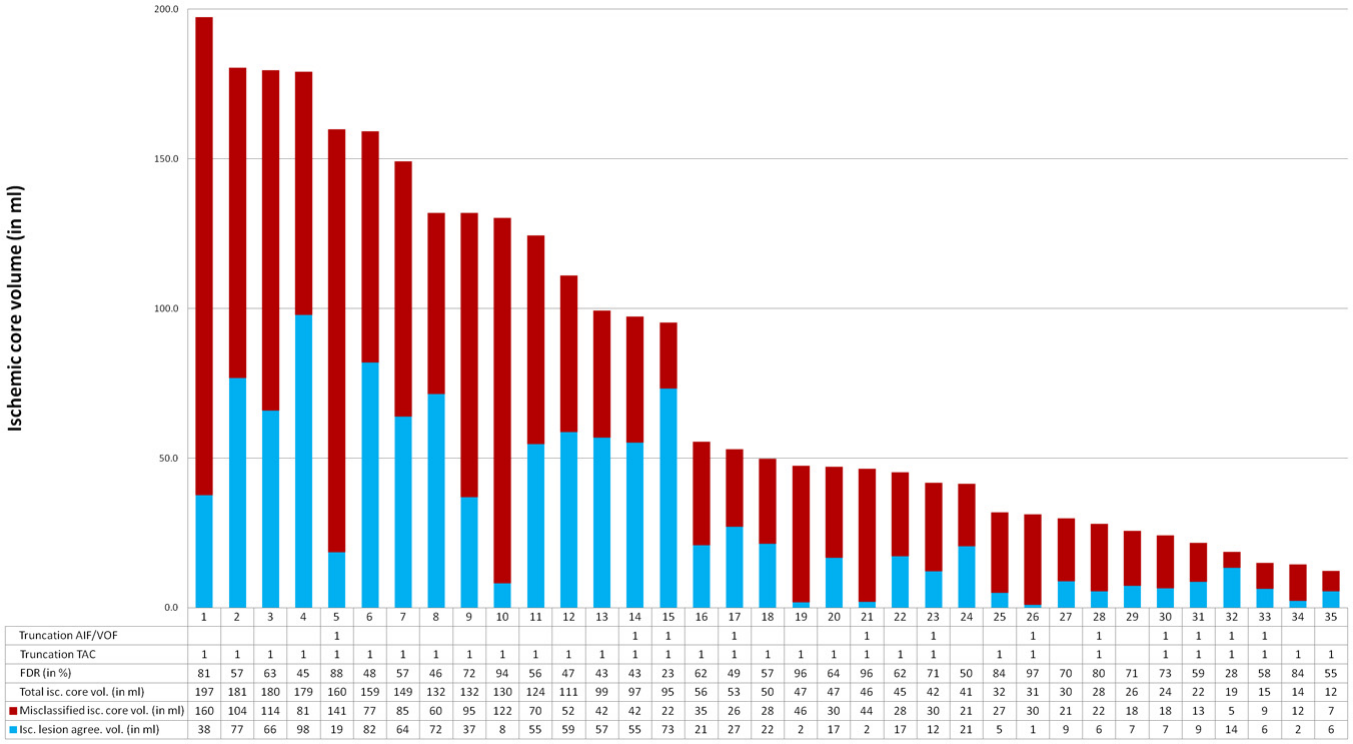
Total ischemic core volume (isc. core vol.), ischemic lesion agreement (light blue) and misclassified ischemic core (dark blue) volume (ml) and FDR (%) for all patients. If truncation was observed in time-attenuation curves (AIF/ VOF or tissue TAC), this is denoted as 1.

Median total CTP ischemic core volume was 49.7ml (IQR: 29.9–131.8ml, min.-max.: 12.2–197.4ml). Median misclassified ischemic core volume was 30.4ml (IQR: 20.9–77.0ml, min.-max.: 5.1–159.7ml). Median ischemic lesion agreement volume was 18.6ml (IQR: 6.5–56.9ml, min.-max.: 1.9–98.0ml). Median FDR was 62% (IQR: 49%-80%, min.-max.: 23%-97%). These values are schematically presented in Fig 2.

Truncation of AIF or VOF was observed in 12 out of 35 patients. Truncation of tissue TAC only was observed in 20 out of 35 patients.

### Perfusion parameter value analysis

**Fig 3:**
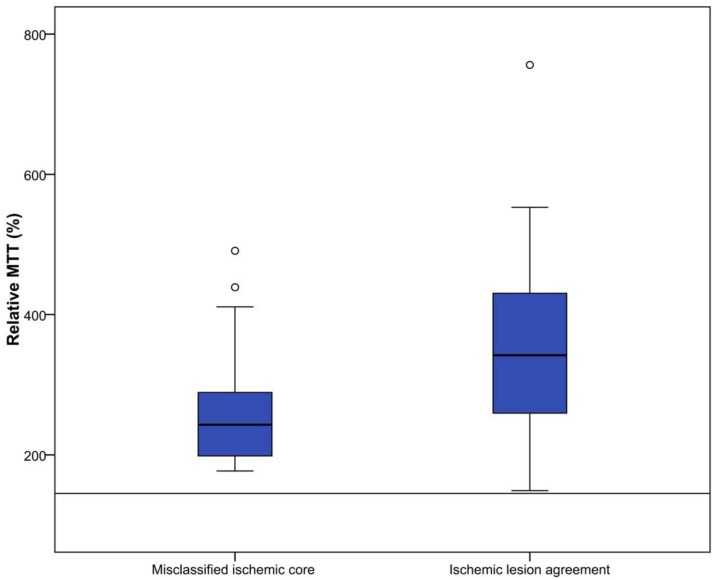
Relative MTT (%) for misclassified ischemic core and ischemic lesion agreement. Threshold value for defining ischemic core is 145%, which is visualized in this figure by the horizontal line.

**Fig 4:**
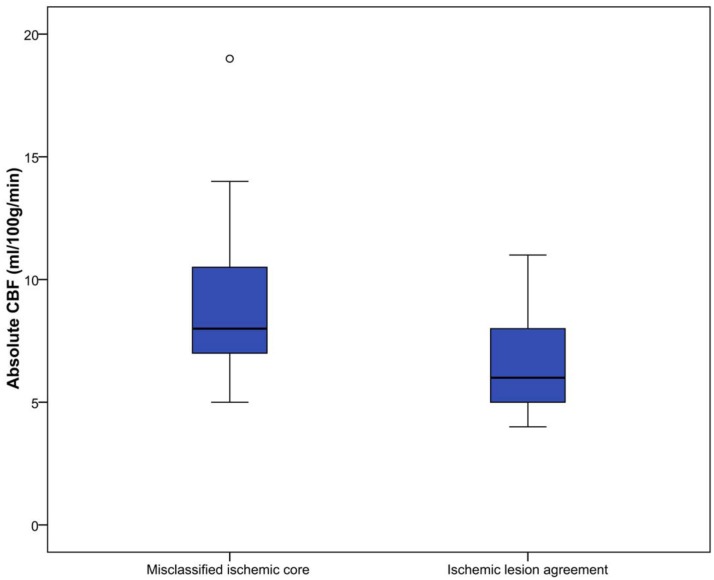
Absolute CBF (ml/100g/min) for misclassified ischemic core and ischemic lesion agreement.

**Fig 5:**
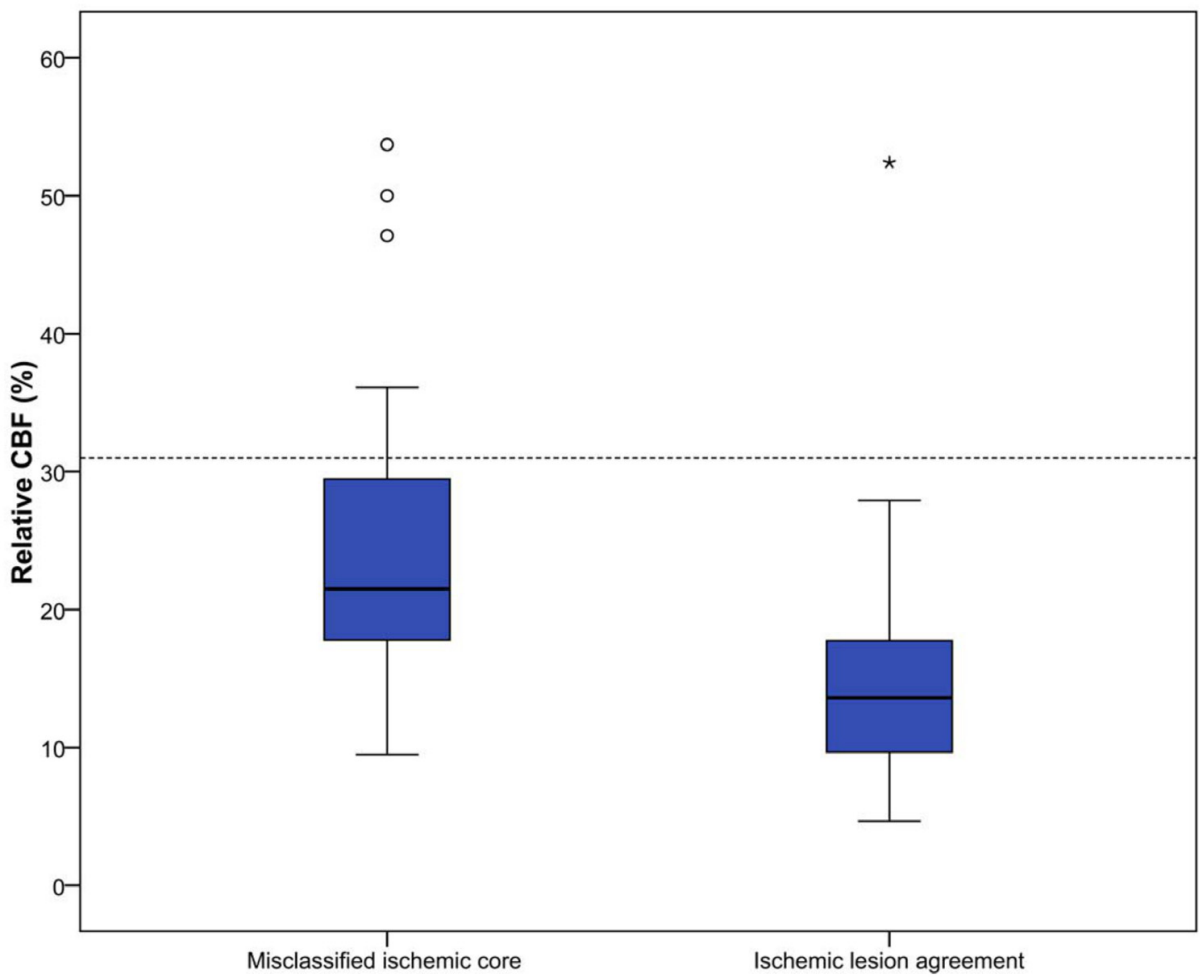
Relative CBF (%) for misclassified ischemic core and ischemic lesion agreement. Proposed hypothetical threshold value for defining ischemic core is 31%, visualized by a dotted line [42].

**Fig 6:**
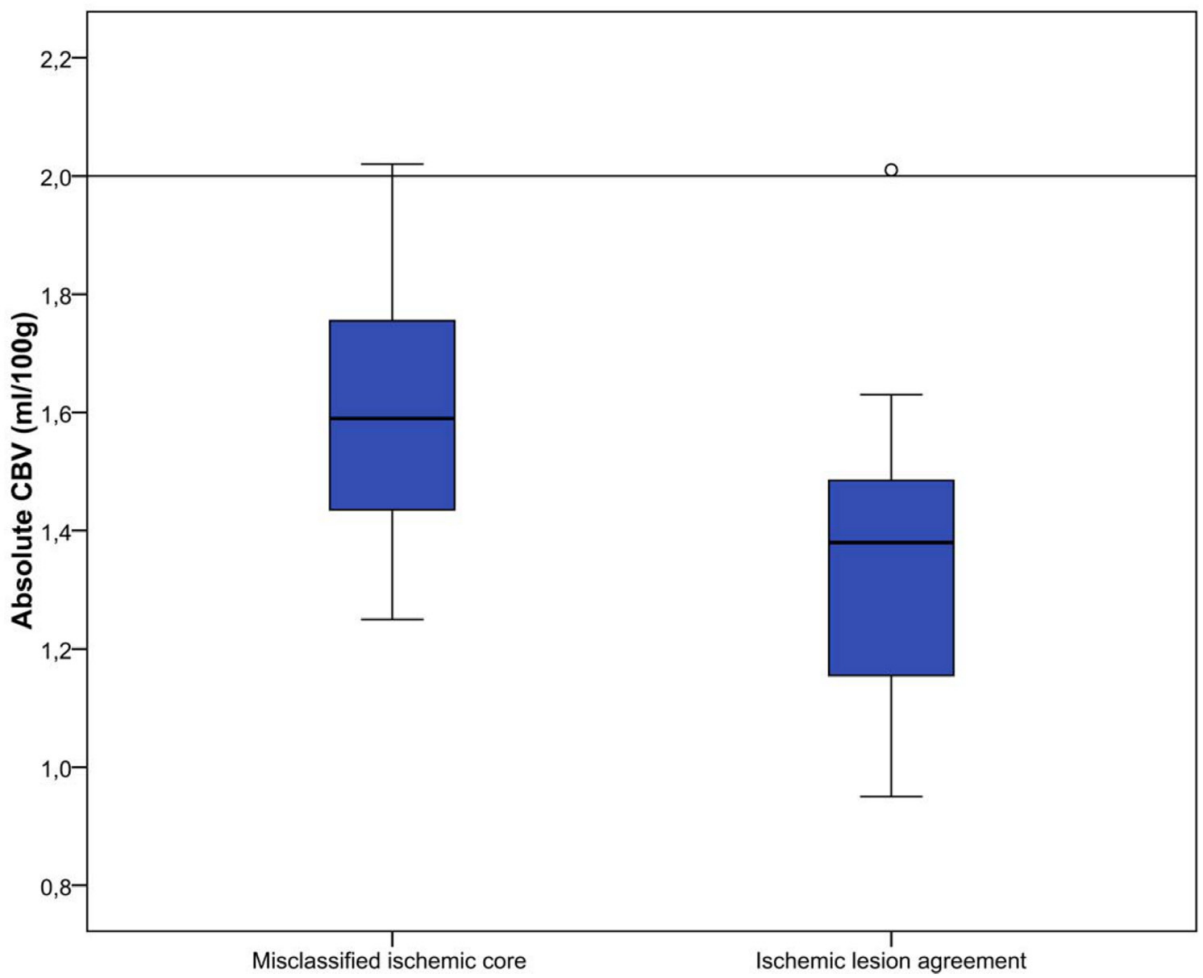
Absolute CBV (ml/100g) for misclassified ischemic core and ischemic lesion agreement. Current threshold value for defining ischemic core is 2.0 ml/100g, and visualized by a horizontal line.

**Fig 7:**
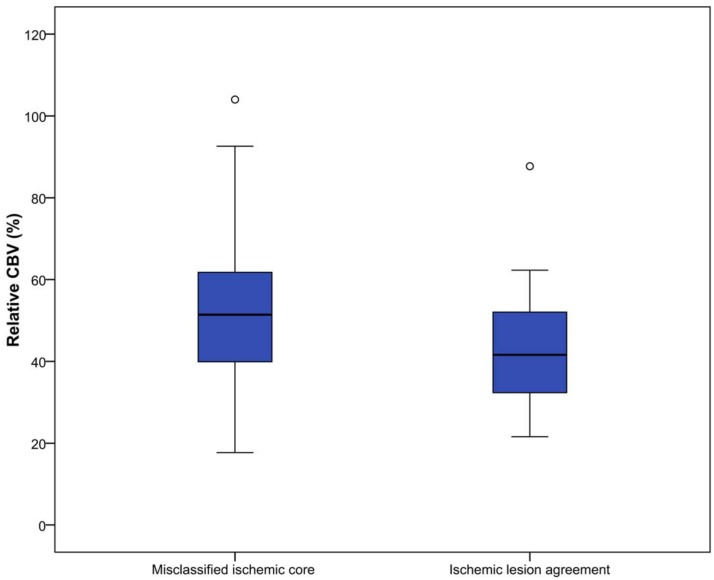
Relative CBV (%) for misclassified ischemic core and ischemic lesion agreement.

All perfusion parameter values were statistically significant different between misclassified ischemic core and ischemic lesion agreement areas(P<0.01). Median parameter values for all patients are shown in Table 2 and Figs 3–7.

**Table 2:** Median perfusion parameter values for misclassified and ischemic lesion agreement of all 35 patient specific values.

Parameter	Misclassified ischemic core median (IQR)	Ischemic lesion agreement median (IQR)
**rMTT** *	243% (198%-289%)	342% (249%-432%)
**aCBF** *	8 ml/100g/min (7–11 ml/100g/min)	6 ml/100g/min (5–8 ml/100g/min)
**rCBF** *	21.5% (17.3%-29.5%)	13.6% (9.7%-18.0%)
**aCBV** *	1.59 ml/100g (1.43–1.79 ml/100g)	1.38 ml/100g (1.15–1.49 ml/100g)
**rCBV** *	51.4% (39.3%-62.0%)	41.6% (31.6%-52.3%)

* Significantly different for misclassified and ischemic lesion agreement region (rank-sum, P<0.01).

## Discussion

Comparison of baseline CTP ischemic core with follow-up noncontrast CT infarct showed that a large part of the ischemic CTP core volume was misclassified. For all perfusion parameters a significant difference was observed between misclassified ischemic core and ischemic lesion agreement. This suggests that currently used thresholds and software used in this study cannot reliably identify ischemic core and may benefit from revisions.

To our knowledge, misclassification of CTP ischemic core volume has not been compared with follow-up noncontrast CT infarct volume to this extent. Several studies have compared CTP ischemic core volume with MR-DWI ischemic core volume obtained at baseline. A high measurement variability in ischemic core volumes was shown between both modalities, with large differences for CTP derived ischemic volumes compared to MR-DWI ischemic core volumes [36,37]. Our study agrees with the limited accuracy of CTP ischemic core volume estimations and shows that ischemic core misclassifications are large. Underlying parameter values differed significantly between misclassified ischemic core and ischemic lesion agreement regions, suggesting room for improvement in threshold definition for ischemic core.

We acknowledge this study comprises a number of limitations. Infarct growth in the time between obtaining baseline CTP and follow-up noncontrast CT can impede accurate comparison between baseline CTP ischemic core volume and follow-up infarct volume. This can lead to underestimation of the FDR of ischemic core on CTP. While misclassified ischemic core can only be caused by flaws in the CTP analysis, agreement between CTP and follow-up noncontrast CT may be partly caused by infarct core growth. The infarcted lesion may grow in the time between baseline and follow-up imaging, therefore it is possible that tissue classified as ischemic core on baseline CTP was actually misclassified at baseline but this misclassification was covered up due to infarct core growth [2,32,33,38].

Strict inclusion criteria were applied, excluding many patients, so results may not be generally applicable. A large number of patients were excluded because of missing follow-up imaging. This may have resulted in a bias of our results since, for example, patients who did not survive the first days could not be included.

The level of misclassification may also be influenced by various baseline and imaging factors and may be correlated with patient outcome. However, with the relative low number of patients included in this study, the statistical power was not sufficient to correctly perform statistical tests to address this relation.

Though our sample size is relatively small, all perfusion parameters showed a significant difference between the two groups. CT imaging was obtained from two manufacturers which could provide a limitation. It is possible that imaging data derived from different scanners produce different results and may not co-operate optimally with the software. However, we observed similar FDR for both scanners. Furthermore, it has been suggested that delay-insensitive methods might produce more accurate ischemic core classifications compared to delay-sensitive methods we used in this study [39]. This however, has recently been disputed. Delay-sensitive methods produce similar ischemic core volumes as delay-insensitive methods if validated thresholds are used [40]. Thresholds used in this study have been validated in previous studies and are currently used by this software in clinical practice. Several other studies found that usage of (sets of) other thresholds for the definition of infarct core used in other software packages might result in more accurate CTP assessments [18,41–44]. Due to limitations of the software we were not able to evaluate the accuracy of using these alternative thresholds to define ischemic core.

Low signal to noise ratio contributes largely to limitation of CTP accuracy [45]. When quantified, CTP showed a relative low contrast-to-noise ratio (CNR), making it problematic to accurately analyze data [36]. Besides that, it has been shown that truncation of time-attenuation curves can cause inaccuracies in CTP-derived hemodynamic measurements [21,46]. We observed truncation in almost all patients. This was largely due to the applied scan protocol of 60 seconds of scanning, as a result of restricted acquisition times. However, we did not find a clear correlation between truncation and FDR, and could thus not relate truncation to inaccuracies in CTP analysis.

Potential causes of the presented errors in CTP analysis could include biological and anatomical causes that can lead false perfusion results and either mimic or hamper (in-)correct diagnosis of acute ischemic stroke. This could be due to certain vascular or neurological diseases, variations in vascular anatomy and dysregulation, upstream flow restriction, cardiac output or lesions and old infarctions [22,23,27,46]. Misplacement of the patient and motion within the scanner, which is likely to occur in emergency settings, can further impede ideal CT imaging [24,27].

CTP software used in this study calculates ischemic core based on pre-set thresholds (rMTT ≥ 145%, aCBV < 2.0 ml/100g) [25]. Median values for both rMTT and aCBV for misclassified ischemic core regions were closer to their threshold values than for ischemic lesion agreement regions, suggesting that some misclassification of ischemic core could be avoided if thresholds would be redefined. Thresholds currently used in CTP analysis are optimized for standard acquisition time (<60 sec). It is shown that extension of acquisition time could produce more accurate results, which is increasingly performed [21,25,46]. Therefore, thresholds used currently could be suboptimal in individual cases. It has been suggested to abandon absolute thresholds and use rCBF as a possible optimal perfusion parameter for assessing ischemic core [42–44]. Our results for the rCBF parameter agree with this novel definition for ischemic core. In some cases usage of rCBF as threshold for ischemic core definition would have excluded some misclassified ischemic core regions.

The approach in which CTP parameters are associated with infarct core can be used to improve the current CTP analysis methods as it gives direct measures of these parameters at locations that end up as final infarct volume. Our study indeed shows that on a group level there are large differences between CTP parameters in CTP ischemic core volumes that end up as infarct and the volumes that ended up as healthy tissue on follow-up imaging. However, on a patient-specific level, current CTP analysis methods are insufficient. Approaches that use thresholding of voxel-based values to determine summary maps of ischemic core and penumbra are limited since CTP only reflects the current state of hemodynamics in brain tissue. Brain tissue’s accumulation of ischemic injury is gradual, and becomes irreversible only if reperfusion is not achieved and after a given time has elapsed. Treatment and time to reperfusion is not included in current CTP analysis packages, which is a conceptual limitation of the method currently used.

CTP can provide a valuable tool for ischemic core volume estimations in acute ischemic stroke patients, if causes for misclassification can be identified. Novel methods to define ischemic core, improved CT scanners, and more accurate postprocessing may improve CTP analysis and may eventually provide a powerful diagnostic method in acute ischemic stroke management.

## Conclusion

This study has shown that perfusion analysis results in a large absolute and relative misclassified ischemic core volume. There was a statistically significant difference for all perfusion parameter values between misclassified ischemic core and ischemic lesion agreement. This suggests that currently used thresholds and software used in this study cannot reliably identify ischemic core and may benefit from revisions.

## References

[1] Wintermark M, Albers GW, Broderick JP, Demchuk AM, Fiebach JB, Fiehler J, et al Acute Stroke Imaging Research Roadmap II. Stroke. 2013;44: 2628–39. 23860298 10.1161/STROKEAHA.113.002015 PMC4040226

[2] Saver JL. Time is brain—Quantified. Stroke. 2006;37: 263–266. 16339467 10.1161/01.STR.0000196957.55928.ab

[3] Jauch EC, Saver JL, Adams HP, Bruno A, Connors JJB, Demaerschalk BM, et al Guidelines for the early management of patients with acute ischemic stroke: A guideline for healthcare professionals from the American Heart Association/American Stroke Association. Stroke. 2013;44: 870–947. 23370205 10.1161/STR.0b013e318284056a

[4] Hacke W, Kaste M, Bluhmki E, Brozman M, Davalos A, Guidetti D, et al Thrombolysis with Alteplase 3 to 4.5 Hours after Acute Ischemic Stroke. N Engl J Med. 2008;359: 877–889. 10.1056/NEJMoa0804656 18815396

[5] Berkhemer OA, Fransen PSS, Beumer D, van den Berg LA, Lingsma HF, Yoo AJ, et al A Randomized Trial of Intraarterial Treatment for Acute Ischemic Stroke. N Engl J Med. 2014; 141217070022009. 10.1056/NEJMoa1411587 25517348

[6] Campbell BCV, Mitchell PJ, Kleinig TJ, Dewey HM, Churilov L, Yassi N, et al Endovascular Therapy for Ischemic Stroke with Perfusion-Imaging Selection. N Engl J Med. 2015; 150211090353006. 10.1056/NEJMoa1414792 25671797

[7] Goyal M, Demchuk AM, Menon BK, Eesa M, Rempel JL, Thornton J, et al Randomized Assessment of Rapid Endovascular Treatment of Ischemic Stroke. N Engl J Med. 2015; 1019–1030. 10.1056/NEJMoa1414905 25671798

[8] Jovin TG, Chamorro A, Cobo E, de Miquel MA, Molina CA, Rovira A, et al Thrombectomy within 8 Hours after Symptom Onset in Ischemic Stroke. 2015; 1–11. 10.1056/NEJMoa1503780 25882510

[9] Saver JL, Goyal M, Bonfare A, Diener H-C, Levy EI, Pereira VM, et al Stent-Retriever Thrombectomy after Intravenous t-PA vs. t-PA Alone in Stroke. 2015; 1–11. 10.1056/NEJMoa1415061 25882376

[10] Yoo AJ, Leslie-Mazwi TM, Jovin TG. Future directions in IAT: better studies, better selection, better timing and better techniques. J Neurointerv Surg. 2013;5 Suppl 1: i1–6. 23572460 10.1136/neurintsurg-2013-010741

[11] Tarpley J, Franc D, Tansy AP, Liebeskind DS. Use of perfusion imaging and other imaging techniques to assess risks/benefits of acute stroke interventions. Curr Atheroscler Rep. 2013;15: 336 23666875 10.1007/s11883-013-0336-6 PMC3683532

[12] Wintermark M, Maeder P, Thiran J-P, Schnyder P, Meuli R. Quantitative assessment of regional cerebral blood flows by perfusion CT studies at low injection rates: a critical review of the underlying theoretical models. Eur Radiol. 2001;11: 1220–1230. 10.1007/s003300000707 11471616

[13] Konstas A, Goldmakher G, Lee T-Y, Lev M. Theoretic basis and technical implementations of CT perfusion in acute ischemic stroke, part 2: technical implementations. AJNR Am J Neuroradiol. 2009;30: 885–92. 19299489 10.3174/ajnr.A1492 PMC7051660

[14] Konstas A, Goldmakher G, Lee T-Y, Lev M. Theoretic basis and technical implementations of CT perfusion in acute ischemic stroke, part 1: Theoretic basis. AJNR Am J Neuroradiol. 2009;30: 662–8. 19270105 10.3174/ajnr.A1487 PMC7051780

[15] Wintermark M, Meuli R, Browaeys P, Reichhart M, Bogousslavsky J, Schnyder P, et al Comparison of CT perfusion and angiography and MRI in selecting stroke patients for acute treatment. Neurology. 2007;68: 694–7. 17325279 10.1212/01.wnl.0000255959.30107.08

[16] Grand S, Tahon F, Attye A, Lefournier V, Le Bas J-F, Krainik A. Perfusion imaging in brain disease. Diagn Interv Imaging. Elsevier Masson SAS; 2013;94: 1241–57. 10.1016/j.diii.2013.06.009 23876408

[17] Song SS. Advanced imaging in acute ischemic stroke. Semin Neurol. 2013;33: 436–40. 24504605 10.1055/s-0033-1364214

[18] Campbell BCV, Yassi N, Ma H, Sharma G, Salinas S, Churilov L, et al Imaging selection in ischemic stroke: feasibility of automated CT-perfusion analysis. Int J Stroke. 2015;10: 51–54. 10.1111/ijs.12381 25319251

[19] Fahmi F, Marquering HA, Streekstra GJ, Beenen LFM, Velthuis BK, VanBavel E, et al Differences in CT perfusion summary maps for patients with acute ischemic stroke generated by 2 software packages. AJNR Am J Neuroradiol. 2012;33: 2074–80. 22555577 10.3174/ajnr.A3110 PMC7965595

[20] Kudo K, Sasaki M, Yamada K, Momoshima S, Utsunomiya H, Shirato H, et al Differences in CT Perfusion Maps Generated by Different Commercial Software : Quantitative Analysis by Using Identical Source. Neuroradiology. 2010;254: 200–209. 10.1148/radiol.254082000 20032153

[21] Borst J, Marquering HA, Beenen LFM, Berkhemer OA, Dankbaar JW, Riordan AJ, et al Effect of Extended CT Perfusion Acquisition Time on Ischemic Core and Penumbra Volume Estimation in Patients with Acute Ischemic Stroke due to a Large Vessel Occlusion. PLoS One. 2015;10: e0119409 10.1371/journal.pone.0119409 25789631 PMC4366202

[22] Lui YW, Tang ER, Allmendinger AM, Spektor V. Evaluation of CT perfusion in the setting of cerebral ischemia: patterns and pitfalls. AJNR Am J Neuroradiol. 2010;31: 1552–63. 20190208 10.3174/ajnr.A2026 PMC7965002

[23] Allmendinger AM, Tang ER, Lui YW, Spektor V. Imaging of stroke: Part 1, Perfusion CT—overview of imaging technique, interpretation pearls, and common pitfalls. AJR Am J Roentgenol. 2012;198: 52–62. 22194479 10.2214/AJR.10.7255

[24] Fahmi F, Beenen LFM, Streekstra GJ, Janssen NY, de Jong HW, Riordan A, et al Head movement during CT brain perfusion acquisition of patients with suspected acute ischemic stroke. Eur J Radiol. Elsevier Ireland Ltd; 2013;82: 2334–41. 10.1016/j.ejrad.2013.08.039 24041432

[25] Wintermark M, Flanders AE, Velthuis B, Meuli R, van Leeuwen M, Goldsher D, et al Perfusion-CT assessment of infarct core and penumbra: receiver operating characteristic curve analysis in 130 patients suspected of acute hemispheric stroke. Stroke. 2006;37: 979–85. 16514093 10.1161/01.STR.0000209238.61459.39

[26] Kaufmann AM, Firlik AD, Fukui MB, Wechsler LR, Jungries CA, Yonas H. Ischemic Core and Penumbra in Human Stroke. Stroke. 1999;30: 93–99. 10.1161/01.STR.30.1.93 9880395

[27] Best AC, Acosta NR, Fraser JE, Borges MT, Brega KE, Anderson T, et al Recognizing False Ischemic Penumbras in CT Brain Perfusion Studies. Radiogr a Rev Publ Radiol Soc North Am Inc. 2012;32: 1179–1196. 10.1148/rg.324105742 22787001

[28] Zhao L, Barlinn K, Bag AK, Kesani M, Cava LF, Balucani C, et al Computed tomography perfusion prognostic maps do not predict reversible and irreversible neurological dysfunction following reperfusion therapies. Int J Stroke. 2011;6: 544–546. 22111800 10.1111/j.1747-4949.2011.00681.x

[29] Campbell BCV, Tu HTH, Christensen S, Desmond PM, Levi CR, Bladin CF, et al Assessing Response to Stroke Thrombolysis: Validation of 24-Hour Multimodal Magnetic Resonance Imaging. Arch Neurol. 2012;69: 46–50. 21911654 10.1001/archneurol.2011.232

[30] Benson J, Payabvash S, Salazar P, Jagadeesan B, Palmer CS, Truwit CL, et al Comparison of CT perfusion summary maps to early diffusion-weighted images in suspected acute middle cerebral artery stroke. Eur J Radiol. Elsevier Ireland Ltd; 2015; 10.1016/j.ejrad.2014.12.026 25623829

[31] Saver JL, Johnston KC, Homer D, Wityk R, Koroshetz W, Truskowski LL, et al Infarct volume as a surrogate or auxiliary outcome measure in ischemic stroke clinical trials. The RANTTAS Investigators. Stroke. 1999;30: 293–298. 9933262 10.1161/01.str.30.2.293

[32] Rosso C, Hevia-Montiel N, Deltour S, Bardinet E, Dormont D, Crozier S, et al Prediction of infarct growth based on apparent diffusion coefficients: penumbral assessment without intravenous contrast material. Radiology. 2009;250: 184–192. 19017923 10.1148/radiol.2493080107

[33] Finitsis S, Kemmling A, Havemeister S, Thomalla G, Fiehler J, Brekenfeld C. Stability of ischemic core volume during the initial hours of acute large vessel ischemic stroke in a subgroup of mechanically revascularized patients. Neuroradiology. 2014;56: 325–332. 24468860 10.1007/s00234-014-1329-z

[34] Klein S, Staring M, Murphy K, Viergever MA, Pluim JPW. elastix: a toolbox for intensity-based medical image registration. IEEE Trans Med Imaging. 2010;29: 196–205. 19923044 10.1109/TMI.2009.2035616

[35] Boers AM, Marquering HA, Jochem JJ, Besselink NJ, Berkhemer OA, van der Lugt A, et al Automated cerebral infarct volume measurement in follow-up noncontrast CT scans of patients with acute ischemic stroke. AJNR Am J Neuroradiol. 2013;34: 1522–7. 23471018 10.3174/ajnr.A3463 PMC8051473

[36] Schaefer PW, Souza L, Kamalian S, Hirsch JA, Yoo AJ, Kamalian S, et al Limited Reliability of Computed Tomographic Perfusion Acute Infarct Volume Measurements Compared With Diffusion-Weighted Imaging in Anterior Circulation Stroke. 2015;M: 419–425. 10.1161/STROKEAHA.114.007117 PMC4308477 25550366

[37] Campbell BCV, Christensen S, Levi CR, Desmond PM, Donnan GA, Davis SM, et al Comparison of computed tomography perfusion and magnetic resonance imaging perfusion-diffusion mismatch in ischemic stroke. Stroke. 2012;43: 2648–2653. 22858726 10.1161/STROKEAHA.112.660548

[38] Zhu G, Michel P, Aghaebrahim A, Patrie JT, Xin W, Eskandari A, et al Prediction of recanalization trumps prediction of tissue fate: The penumbra: A dual-edged sword. Stroke. 2013;44: 1014–1019. 23463751 10.1161/STROKEAHA.111.000229

[39] Konstas AA, Lev MH. CT perfusion imaging of acute stroke: the need for arrival time, delay insensitive, and standardized postprocessing algorithms? Radiology. 2010;254: 22–25. 20032139 10.1148/radiol.09091610

[40] Man F, Patrie JT, Xin W, Zhu G, Hou Q, Michel P, et al Delay-sensitive and delay-insensitive deconvolution perfusion-CT: similar ischemic core and penumbra volumes if appropriate threshold selected for each. Neuroradiology. 2015; 10.1007/s00234-015-1507-7 25749851

[41] Olivot J-M, Mlynash M, Thijs VN, Kemp S, Lansberg MG, Wechsler L, et al Optimal Tmax threshold for predicting penumbral tissue in acute stroke. Stroke. 2009;40: 469–75. 19109547 10.1161/STROKEAHA.108.526954 PMC2670783

[42] Campbell BCV, Christensen S, Levi CR, Desmond PM, Donnan GA, Davis SM, et al Cerebral blood flow is the optimal CT perfusion parameter for assessing infarct core. Stroke. 2011;42: 3435–3440. 21980202 10.1161/STROKEAHA.111.618355

[43] Kamalian S, Kamalian S, Maas MB, Goldmacher G V., Payabvash S, Akbar A, et al CT cerebral blood flow maps optimally correlate with admission diffusion-weighted imaging in acute stroke but thresholds vary by postprocessing platform. Stroke. 2011;42: 1923–1928. 21546490 10.1161/STROKEAHA.110.610618 PMC3125441

[44] Dani KA, Thomas RGR, Chappell FM, Shuler K, MacLeod MJ, Muir KW, et al Computed tomography and magnetic resonance perfusion imaging in ischemic stroke: definitions and thresholds. Ann Neurol. 2011;70: 384–401. 21796665 10.1002/ana.22500

[45] Gonzalez RG. Low signal, high noise and large uncertainty make CT perfusion unsuitable for acute ischemic stroke patient selection for endovascular therapy. J Neurointerv Surg. 2012;4: 242–245. 22679196 10.1136/neurintsurg-2012-010404

[46] Mangla R, Ekhom S, Jahromi BS, Almast J, Mangla M, Westesson PL. CT perfusion in acute stroke: Know the mimics, potential pitfalls, artifacts, and technical errors. Emerg Radiol. 2014;21: 49–65. 23771605 10.1007/s10140-013-1125-9

